# Articular Cartilage Regeneration through Bioassembling Spherical Micro-Cartilage Building Blocks

**DOI:** 10.3390/cells11203244

**Published:** 2022-10-16

**Authors:** Brian E. Grottkau, Zhixin Hui, Yonggang Pang

**Affiliations:** The Laboratory for Therapeutic 3D Bioprinting, Department of Orthopaedic Surgery, Massachusetts General Hospital, Harvard Medical School, 55 Fruit St., Boston, MA 02114, USA

**Keywords:** articular cartilage regeneration, bioassembly, spherical micro-cartilage, building blocks, 3D bioprinting

## Abstract

Articular cartilage lesions are prevalent and affect one out of seven American adults and many young patients. Cartilage is not capable of regeneration on its own. Existing therapeutic approaches for articular cartilage lesions have limitations. Cartilage tissue engineering is a promising approach for regenerating articular neocartilage. Bioassembly is an emerging technology that uses microtissues or micro-precursor tissues as building blocks to construct a macro-tissue. We summarize and highlight the application of bioassembly technology in regenerating articular cartilage. We discuss the advantages of bioassembly and present two types of building blocks: multiple cellular scaffold-free spheroids and cell-laden polymer or hydrogel microspheres. We present techniques for generating building blocks and bioassembly methods, including bioprinting and non-bioprinting techniques. Using a data set of 5069 articles from the last 28 years of literature, we analyzed seven categories of related research, and the year trends are presented. The limitations and future directions of this technology are also discussed.

## 1. Introduction

Articular cartilage defects are prevalent and can result from trauma, osteoarthritis, osteochondrosis dissecans, osteonecrosis, and chronic polyarthritis [1]. In adults, articular cartilage lesions often lead to osteoarthritis, affecting one out of seven adults in America—32.5 million people [2]. Osteoarthritis is the primary cause of pain and disability among US adults. The cases of physician-diagnosed arthritis are estimated to be 78.4 million by 2040 [3]. Articular cartilage is a vascularized tissue, and it does not have the capability to regenerate on its own.

The existing treatments for articular cartilage defects include debridement, microfracture, osteochondral autograft transfer, osteochondral allograft, and autologous chondrocyte implantation [4]. However, these treatments each have limitations and drawbacks. Microfracture only demonstrates short-term pain relief, and therapeutic effects tend to deteriorate over time because of the weak biomechanical nature of the generated fibrocartilage and scar tissues [5]. Autologous osteochondral grafts result in new lesions in the host joint, and osteochondral allografting faces limited donor availability and the risk of transmitted diseases [6].

Tissue engineering is an interdisciplinary field that applies the principles of engineering and the life sciences to develop biological substitutes that restore, maintain, or improve tissue function [7]. Cartilage regeneration was one of the first research fields in tissue engineering [8]. Engineered cartilage has been used in clinical practice, such as matrix-assisted autologous chondrocyte implantation (MACI) [9]. This approach utilizes a biopsied joint cartilage tissue to harvest and expand chondrocytes in vitro and reimplant them to the cartilage defect with a hydrogel matrix. This technique has the drawbacks of limited cell number, poor cell quality (chondrocytes harvested from the aged or injured autologous cartilage), non-hyaline cartilage growth and non-integrated implants in some situations. Conventionally, most existing cartilage tissue engineering methods in preclinical or clinical applications, including MACI, use a top-down approach. This technique usually seeds chondrocytes or stem cells in a bulk-sized scaffold or matrix with the same or similar dimensions as the targeted cartilage tissue.

In contrast, there is a bottom-up approach, also known as bioassembly, defined as “the fabrication of hierarchical or hybrid cell–material constructs with a prescribed 2D or 3D organization through automated assembly of pre-formed cell-containing fabrication units generated via cell-driven self-organization or the use of bottom-up fabrication technologies” [10].There are different types of building blocks that can be used including cell sheets [11], organoids [12], cellular aggregates/spheroids [13], tissue strands [14], and rings [15]. Various methods have also been reported to generate building blocks. Different bioassembly methods have also been utilized, such as simple pipetting, pipetting into molds, and bioprinting methods.

In this review, we focused on spherical micro-cartilage building blocks that are micro-constructs containing live chondrogenic cells, including chondrocytes and mesenchymal stem cells, for articular cartilage regeneration. Depending on whether building blocks consist of biomaterials, they are categorized into multiple cellular scaffold-free spheroids, and cell-laden polymer and hydrogel microspheres.

## 2. Definition of Bioassembly

In the literature, the concept of bioassembly generally refers to combining small bio-units to form a larger unit. The types of bio-units are very broad, including cells [16,17,18], enzymes [19], aerogels [20], nanotubes [21], and even quantum dots [22]. Here we use the concept of bioassembly specifically as “facilitates the automated assembly of cell containing building blocks, where the minimum fabrication units are preformed cell-containing building blocks with sizes large enough so that automated assembly can technologically be achieved” [10,23].

## 3. Advantages of Bioassembly Compared with Top-Down Approaches

(1) In a macroscale scaffold, it is challenging to seed cells with a uniform distribution and at densities that match the corresponding native tissues. Low cell densities and heterogeneous spatial cell distributions often occur in macro-sized scaffolds because it is difficult to immobilize live cells in scaffolds. In contrast, micro-building blocks do not have these issues because of sub-millimeter sizes.

(2) It is also complicated to generate spatially organized multicellular structures or complex tissue features in macro-sized constructs, such as repeated features or structures, tissue junctions, tissue interfaces, and structural zonations. In contrast, micro-building blocks with different tissue types can be generated individually and spatially assembled afterwards.

(3) Scaffold-based approaches generally fail to mimic the unit-repetitive modular patterns that exist in native human tissues, such as nephrons, lobules, and islets. In contrast, it is easier to generate numerous replicates of the building blocks to represent the repetitive units.

(4) Top-down approaches usually have a higher cost than bottom-up approaches in scaled-up production.

## 4. Spherical Building Blocks Manufacture

Depending on whether consisting biomaterials, spherical building blocks are typically divided into scaffold-free multiple cellular spheroids (cellular aggregates), cell-laden polymer microspheres, and cell-laden hydrogel microspheres.

### 4.1. Scaffold-Free Cellular Spheroids

**Figure 1:**
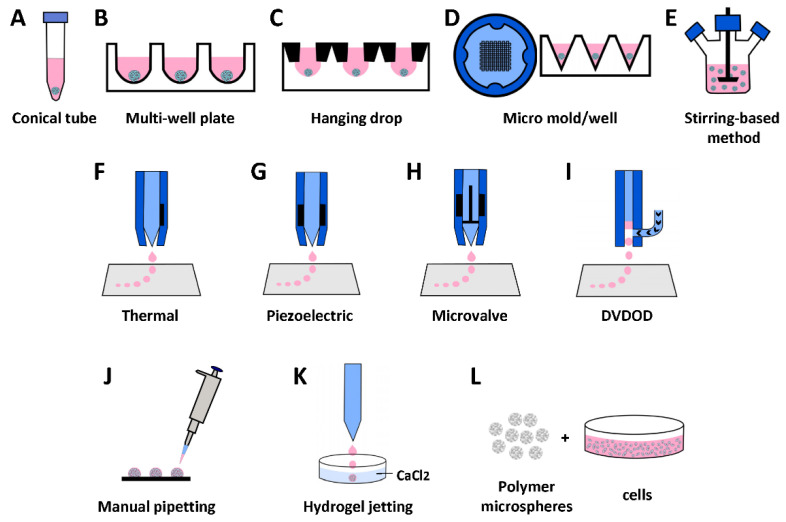
Illustration of common methods for generating spherical building blocks. (**A**) Conical tube or deep-well plates centrifugation. (**B**) Round bottom non-adhesion plate method. (**C**) Hanging drop method. (**D**) Micro-mold/well method. (**E**) Stirring-based method. (**F**–**I**) 3D droplet bioprinting methods: (**F**) Thermal actuation-based inking jet bioprinting. (**G**) Piezoelectric actuation-based inking jet bioprinting. (**H**) Microvalve-based bioprinting. (**I**) Direct-volumetric drop-on-demand (DVDOD) bioprinting. (**J**) Manual pipetting. (**K**) Hydrogel jetting (for alginate microspheres). (**L**) Polymer microspheres.

**Figure 2:**
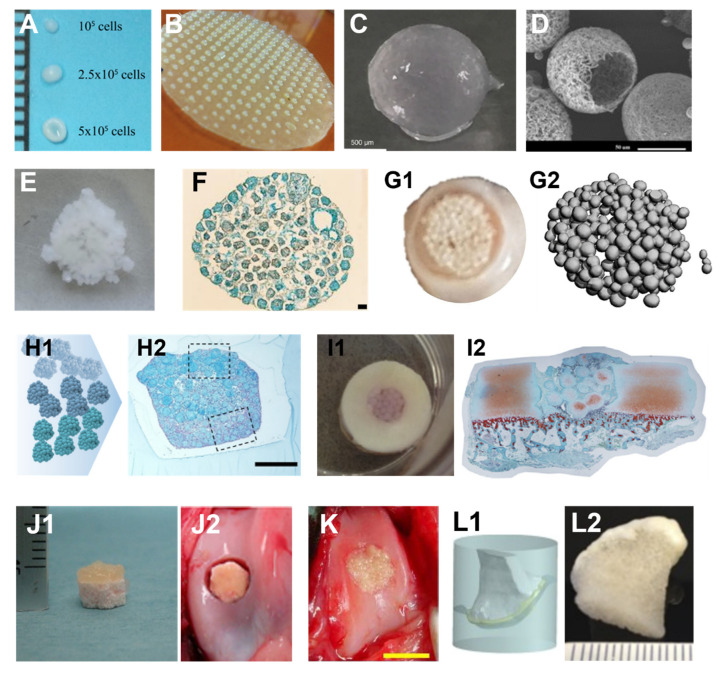
Examples of cartilage spherical building blocks generated and bioassembled using non-bioprinting methods. (**A**) Typical images of mesenchymal stem cell spheroids generated using the deep-well centrifugation methods. The number of cells per well affects the size and shape of a spheroid. (**B**) Microwell method and generated spheroids. (**C**) A hydrogel microsphere generated using alginate and chondrocytes. (**D**) An SEM image of nanofibrous polymer microsphere. (**E**) An assembled macro-tissue by bioassembling alginate/chondrocyte microspheres and individual microspheres are still visible. (**F**) A histological image of an assembled macro-tissue using chondrocyte spheroids. (**G**) A macroscopic image of chondrocyte spheroids after 8 weeks of manual bioassembly process in a well (**G1**) and individual spheroids are still visible, shown by micro-CT imaging (**G2**). (**H**) Spheroids generated using three different types of cells (**H1**) can be fused together into a zonal structure (**H2**, histological staining) using the manual bioassembly method. (**I**–**K**) Examples of bioassembly in osteochondral defect repair. (**I**) Spheroids manual bioassembly in an in vitro osteochondral defect model (**I1**: macroscopic view; **I2**: microscopic view of histological staining) (**J**) In vivo osteochondral defect repairing (**J2**) using an osteochondral graft (**J1**) generated by bioassembling cell-laden hydrogel microspheres on top of a bone-like scaffold or using only chondrocyte spheroids (**K**). (**L**) An example of anatomically shaped cartilage (**L2**) generation using a custom-designed mold (**L1**). Spheroids were placed on top of a pre-milled bone graft inside the mold (Figures adapted from [12,16,24,27,28,29,30,31,32,33]).

Cell aggregating is one of the earliest stage methods to generate scaffold-free cellular spheroids. One simple approach is to add cell suspension in a conical tube (Figure 1A). After centrifuging, cells aggregate to form a pellet—a large spheroid [24]. This method usually forms large pellets (diameter > 1 mm), which are less spherical than the ones in other methods (Figure 2A). Seeding cells in multiple-well plates (Figure 1B) with non-adhesion surface or specially designed hanging drop (Figure 1C) configuration generates more uniform and smaller spheroids in higher throughput. A microwell-plate-based method (Figure 1D) has been reported, aimed at generating even smaller spheroids (diameter < 100 um) for better spheroid fusion [16] (Figure 2B). Various stirring-based methods have been reported to generate spheroids in larger quantities [25], including spinner flask (Figure 1E), dynamic suspension bioreactor, rotating wall vessel bioreactor, and Taylor vortex flow bioreactor [26]. Two major drawbacks of the stirring method are (1) apparent non-uniform sizes and (2) shear-force-induced cell damage during the stirring.

### 4.2. Cell-Laden Microspheres

#### 4.2.1. Cell-Laden Polymer Microsphere

The first step of this technique is to generate porous microspheres (Figure 2D) using biocompatible and bioabsorbable polymers. There are various reported methods to generate porous microspheres [34,35], including solvent evaporation, polymerization, seed swelling, sinter, synthesis, phase separation, and spray-drying methods. The second step is to seed cells into the microspheres and culture them for a specific period (Figure 1L) [28,35].

#### 4.2.2. Cell-Laden Hydrogel Microsphere

The methodology for generating cell-laden hydrogel microspheres is versatile, including manual pipetting (Figure 1J) [36], cell–hydrogel emulsification, machine-driven hydrogel-microsphere jetting, and various bioprinting technologies (Figure 1F–I).

##### Hydrogel Jetting

One of the most common methods is to dispense a stream of droplets of alginate hydrogel solution-containing cells into a calcium solution bath (Figure 1K). The droplets solidify in the bath, and the cells are entrapped inside the alginate spheres (Figure 2C). Standard methods to stream alginate droplets include coaxial air-flow, electrostatic, vibration, and mechanical cutting [37]. One advantage of this method is that large quantities of cell-laden hydrogel microspheres can be generated in a timely manner. The drawbacks of hydrogel jetting include minimal types of hydrogel being applicable, and relatively low cell concentration can be achieved. More importantly, without chemical modification alginate, hydrogel lacks binding sites for cell attachment, resulting in limited cell spreading and impaired cell function, which makes it challenging to fuse cell-encapsulated alginate microspheres (Figure 2E).

##### Bioprinting Cell-Laden Hydrogel Microspheres

Bioprinting is generally considered to be the use of computer-aided transfer processes for patterning and assembling living and non-living biomaterials with a prescribed 2D or 3D organization to produce bioengineered structures serving in regenerative medical, pharmacokinetic, and cell biological studies [38]. The materials used in bioprinting include molecules, cells, tissues, and biodegradable biomaterials [39]. Several bioprinting techniques can be utilized to generate cell-laden hydrogel microspheres.

Generally speaking, there are two types of techniques to bioprint microspheres according to how the volumes of the bioinks are controlled: (1) indirect volumetric control, including inkjet (Figure 1F,G) and microvalve (Figure 1H), and (2) direct volumetric control: DVDOD (Figure 1I) [40]. The volumes of droplets in indirect method cannot be controlled mechanically and they are controlled indirectly by other parameters, and the volumes of droplets are calculated after calibrations. The direct method mechanically controls the plunger of a dispensing syringe, the volumes of droplets are preassigned without being affected by other parameters, and a calibration is usually not required.

(i) Inkjet bioprinting

Inkjet bioprinting takes a similar ‘inkjet’ approach used in a desktop ‘office’ inkjet printer. Some early works of bioprinting were actually accomplished by directly using the hardware of a desktop inkjet printer. A mixture of cells and hydrogel were loaded to the ‘cleaned and sterilized’ ink cartridge and deposited dropwise [41]. For better control and performance, various custom-made inkjet bioprinters have been reported, with most using thermal (Figure 1F) or piezoelectric (Figure 1G) actuator-driven droplet techniques [42]. The volume of droplets generated by a thermal actuator can be altered by varying the temperature gradient or by increasing the frequency of the pressure pulses. Inkjet bioprinting can dispense small volume droplets (per droplet down to picoliter level), which generates better precision in theory. There is a tradeoff of the small volume droplet because it can dry up quickly on the substrate, which is unfavorable for live-cell bioprinting. Furthermore, this technology is usually only applicable for a bioink of low viscosity. The small diameter nozzle may generate sheer forces harmful to cells and the volume of droplets can only be indirectly controlled.

(ii) Microvalve-based bioprinting

The microvalve-based dispensing system is usually equipped with a pressurized bioink container and distal end micro-solenoid valve [43]. By controlling the open duration and the degree of pressure applied to the bioink container, the volume of the droplets can be controlled. The microvalve-based dispending system can usually dispense bioink with higher viscosity but dispenses a larger volume per droplet than inkjet bioprinting. Another advantage of the microvalve-based bioprinting technique is that its hardware costs are relatively lower than those of inkjet bioprinting.

(iii) Direct volumetric bioprinting

As we documented above, the droplet volume in inkjet and microvalve bioprinting is indirectly controlled. When bioink changes, the droplet volume changes, even though the same bioprinting parameters are used. Therefore, to control the volume of droplets of bioink, calibration is needed for each bioprinting batch. Furthermore, the viscosity of the bioink may fluctuate during the same assay when cells condensate. To overcome this limitation, a direct volumetric drop-on-demand technology was invented (Figure 1I) [40] that has two unique features: (1) dispensing a specific volume of bioink (such as 1 nl) to the dispensing nozzle using a linear actuator. This mechanism enables accurate volumetric dispensing, regardless of the viscosity of the bioink. (2) The bioink is then dispensed out of the nozzle in a non-contact manner by pulsed air. For single droplet dispensing, the pulsed air can be set to the low-pressure mode that the pressure only provides a dispensing force. In a multiple-droplet dispensing mode, when multiple droplets form a single hydrogel microsphere on a substrate, the dispensing pressure can be deliberatively selected to low or high pressure so that the colliding forces between droplets can alter the pattern of cell distribution inside. This technique can be used to generate microtissues with specific cellular patterns, which in turn control cell functions.

## 5. Bioassembly Methods

We here summarize the bioassembly methods into two categories, bioprinting and non-bioprinting methods, based on whether bioprinting technology is utilized during the assembly process (Table 1 and Table 2).

### 5.1. Manual Assembly

#### 5.1.1. Simple Pipetting

The most widely used method for building block bioassembly is manual operation. This can be simply pipetting the building blocks to any confined spaces, such as a well of a multiple-well plate. The building blocks can fuse together because of close contact with each other (Figure 2E–G). Multiple tissue types of building blocks can be assembled into a macro-tissue with more complex structures. For example, an osteochondral graft-like tissue can be formed by plating a few layers of chondral spheroids over several layers of osteogenic spheroids (Figure 2H) [89]. In addition, spheroids can be directly pipetted into an osteochondral defect in an in vitro model (Figure 2I1) or in vivo (Figure 2K). From the histology of the in vitro model, cell death and immature cartilage tissue were observed, which needs to be improved in future studies.

#### 5.1.2. Bioassembly in an Anatomically Shaped Mold

Because of their miniature size, the building blocks can well fit an anatomically shaped mold to bioassemble into a macro-tissue with a designed shape. Cartilage-like macro-tissues can be assembled in cylindrical, circular, square, and triangular shapes as a proof of concept using corresponding molds in simple shapes [90]. By bioassembling hydrogel microspheres on top of a cylinder bone-like scaffold (Figure 2J1), an osteochondral graft can be generated to repair an osteochondral defect (Figure 2J2). More complex-shaped engineered cartilage tissues generated using bioassembly were also reported [90]. A piece of bone was milled to the shape of a partial femoral condyle and placed in a silicone mold and the scaffold-free human mesenchymal stem cell (hMSCs) spheroids were placed on top of the bone base (Figure 2L1). An anatomically shaped osteochondral graft was then created after weeks of culture (Figure 2L2) [24]. In another report, an entire joint with a cartilage surface matching that of a rat femoral condyle was created using nanofibrous hollow microspheres loaded with rabbit chondrocytes [35].

### 5.2. Bioassembly Using 3D Bioprinting

#### 5.2.1. Assembly Using Extrusion Bioprinting

**Figure 3:**
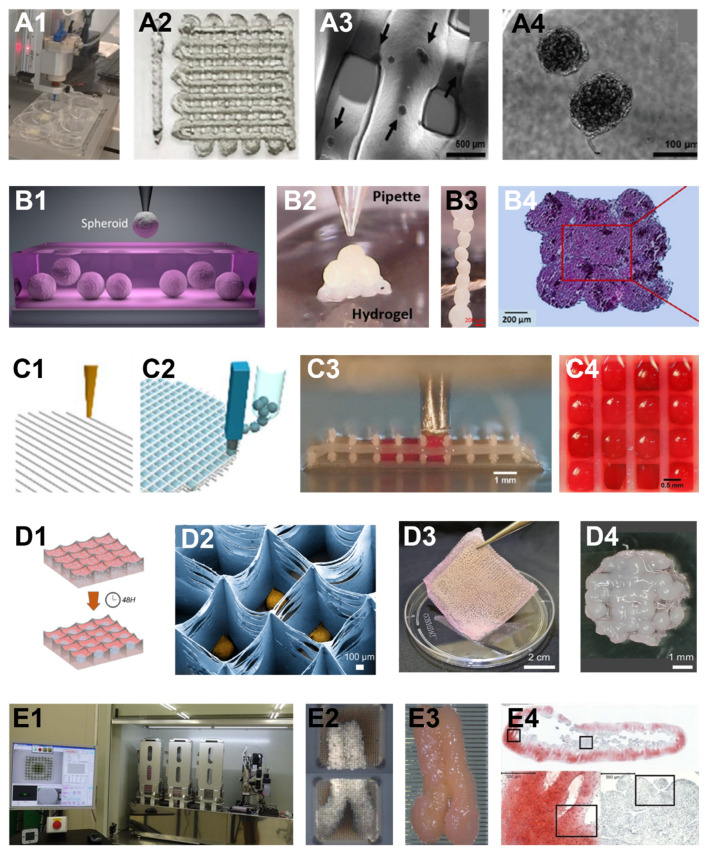
Examples of bioassembly methods using bioprinting. (**A1**–**A4**) Extrusion-bioprinting-based bioassembly. Cellular spheroids were mixed with a hydrogel as bioink and loaded into an extrusion bioprinter (**A1**) to deposit bioink in the format of filaments to a substrate (**A2**–**A4** are zoom-in views). (**B1**–**B4**) Aspiration-assistant bioprinting for bioassembly. An aspiration tip was used to pick up individual spheroids (**B1**) and transfer the spheroids onto a hydrogel surface or into a hydrogel bath. The spheroids were eventually positioned in a predefined pattern (**B2**, **B3**) and fused into a macro-tissue (histology, **B4**). (**C1**–**C4**) Hybrid bioprinting uses two independent nozzles to deposit polymer filaments (**C1**) and spherical building blocks (**C2**). The hydrogel microspheres were deposited into each grid of the polymer filaments (**C3**,**C4**). (**D1**–**D4**) A different hybrid bioprinting method assembled a single layer of spheroids and a polymer slab (**D1**) with shallow chambers into a hybrid graft. MSCs were deposited into the chambers using inkjet bioprinting (**D1**). Spheroids were formed within each chamber (**D2**) and attached to the whole polymer slab (**D3**). A hybrid graft composed of polymer and fused spheroids was eventually formed (**D4**). (**E1**–**E4**) A needle-array-based bioassembly method deposits spheroids into individual needles in a ‘kebab’ way (**E1**: bioprinter, **E2**: spheroids in two needle arrays). The spheroids were fused within the needle array (**E2**). After the fused macro-tissue was removed from the needle array, it further grew and developed (**E3**, a second fusion of two piece of fused tissues). Void spaces, immature cartilage tissue, and cell death were observed (**E4**, histology). (Figures adapted from [17,42,77,80,92,93]).

3D bioprinting has precise deposition, and also demonstrates advantages in building block bioassembly. Multiple cellular spheroids can be loaded into a bioink container (usually a syringe) and directly deposited into another sticky hydrogel [91] or scaffold filaments [17] to be held in place (Figure 3(A1–A4)). This technique requires a relatively large quantity of spheroids in the syringe for smooth dispensing, which can sometimes be wasteful.

#### 5.2.2. Assembly Using Aspiration Bioprinting

To overcome this limitation, an aspiration-based bioprinting technology was reported to pick cellular spheroids individually from a spheroid container (Figure 3B1) to a holding hydrogel (Figure 3(B2,B3)) and fused to a macro-tissue (Figure 3B4) [79,80]. This method requires real-time image processing to identify spheroids individually. It theoretically does not waste any spheroids, and the placement of individual spheroids is accurate because of the high mechanical accuracy in a bioprinting system. However, in reality, the assembled result of aspiration-based bioprinting seems to be less favorable than direct bioprinting. This is because direct bioprinting deposits spheroids continuously, while aspiration-based bioprinting needs multiple movements, generating more disruption to the holding hydrogel that spheroids shift (Figure 3B3). In addition, negative pressure is required to release individual spheroids off the aspiration head, disrupting the previously deposited spheroids. Another limitation of aspiration-based bioprinting is its slowness.

#### 5.2.3. Assembly Using Spherical Building Blocks and Polymer Hybrid Bioprinting

Bioprinting-based bioassembly has another advantage of being able to combine building block bioprinting with polymer bioprinting. For example, immediately after bioprinting a scaffold using polymer filaments (Figure 3C1), hydrogel microspheres can be deposited to the spaces between polymer strands (Figure 3(C2–C4)). In this manner, an anatomically shaped scaffold with incorporated hydrogel microspheres can be generated. This approach can preserve the good mechanical strength of a polymer scaffold and maintain a high cellular density. However, because polymer needs to be printed at high temperatures (from 100 °C to 230 °C), one drawback of this method is thermal stresses from polymer structs resulting cell death [94]. In another example, polymer material was initially used to 3D print a slab consisting of fibrous microchambers (Figure 3(D1–D4)). Cells were then bioprinted into each chamber to grow into spheroids. After in vitro culture, spheroids integrate into the polymer, forming a unique hybrid graft [42].

#### 5.2.4. Needle-Array-Based Spheroid Bioassembly

Similar to aspiration-based bioprinting assay, needle array bioassembly requires aspirating individual cellular spheroids using suction force. The difference is that it places spheroids into needles of an array, and one needle can hold multiple spheroids like a kebab (Figure 3E1) [93,95]. Placing spheroids to specific needles in a controlled manner can form an array of spheroids in a particular shape. The needles are designed to be close enough that spheroids can fuse to each other while in the needle array. One advantage of this method is that the positions of individual spheroids are well-controlled. Global shrinkage is minimal compared with other bioassembly methods because needles can hold spheroids in place. Even though the spaces occupied by needles leave holes in the macro-tissues, the holes can be filled by cells after the macro-tissue is released from the needle array [88]. The obvious downside of this technique is that the shape-resolution of the assembled tissue is low because of the limited number of needles in an array (9 × 9 or 26 × 26). However, multiple needle arrays can be used, and each array constructs a proportion of the targeted tissue to increase the resolution (Figure 3(E2,E3)). The needle array method does not solve the problems of cell death and immature tissue development (Figure 3E4). These problems were also reported in a manual bioassembly study, as we mentioned above (Figure 2(I1,I2)) [16].

## 6. Literature Summary of Bioassembling Micro-Cartilage Building Blocks

To analyze the development of the specific field of research that we focus on in this article as compared with its parent categories, such as bioassembly of any types of tissues and all research on cartilage tissue engineering and bioprinting, we performed literature research using Pubmed. We first searched Pubmed for non-review research articles on “bioassemble micro-cartilage building blocks into macro-cartilage”. We used three categorical keywords and their variant forms: (1) the cartilage-related keywords, such as cartilage and chondrocyte; (2) the building-block-related keywords, such as microtissue, spheroid, pellet, organoid, micro-construct, cell-laden microsphere, cellular aggregate, modular tissues, and chondrosphere; (3) the bioassembly-related keywords, such as building block, module, bottom-up, assembly, bioassembly, fusion, merge, and building unit. The qualified 110 articles must contain all three categorical keywords or variants in their titles, abstracts, or MeSH terms. Finally, all 110 articles were evaluated by two scientists using a full-text review to exclude non-qualified articles. These exclusions include the following situations: (1) The keywords were defined differently from what was used in the current review. For example, bioassembly occurs at biomolecule or cellular instead of microtissue levels. (2) The content of “bioassemble micro-cartilage building blocks into macro-cartilage” is only discussed and not part of the experiment performed. (3) The article is irrelevant in other ways. A total of 59 articles that met the above criteria were finally selected. Among them, 15 articles reported the application of bioprinting and 44 of non-bioprinting for this purpose. The above search results were assigned to three categories: (1) micro-cartilage bioassembly with bioprinting, (2) micro-cartilage bioassembly without bioprinting, and (3) micro-cartilage bioassembly using any technologies. We have seven categories in total, and categories four to seven are (4) bioassembly for any tissue regeneration using the keyword “bioassembly”, (5) cartilage bioprinting using the keyword “cartilage bioprinting”, (6) cartilage tissue engineering accomplished with any technologies using the keyword combination “cartilage AND bioprinting”, and (7) bioprinting application for any tissue regeneration using the keyword “bioprinting”. Multiple keyword filters, as stated above in titles and abstracts were used for categories four to seven to select related articles, and any cancer research-related articles were excluded. In addition, full-text analysis was used for category 4 to remove any irrelevant articles, and 154 articles were eventually included; an abstract review was performed to exclude irrelevant articles in cartilage bioprinting, and 223 articles were included. In total, 3402 cartilage tissue-engineering-related articles and 1590 bioprinting-related articles were found to be relevant and eventually included after multiple keyword filtering. We acknowledge that (1) keyword filtering is less accurate than full-text analysis, and there might potentially be under-included and over-included articles, (2) some articles may be included in more than one category because of the child–parent categorical relationships, or they may fall in more than one parent category. Our study does not include potentially related articles that were not included in the Pubmed database.

We analyzed yearly trends and milestones from the entire available date range of each category. Because some categories are child categories of others, comparing the numbers of publications is less meaningful. We, therefore, use the percentage of articles in the trending analysis. As shown in Table 3, these milestones include: (1) the number of articles in a specific category; (2) the year of the first article in a category was published according to Pubmed using our search protocols; (3) the number of years when the cumulative percentages of articles just reached or passed quartiles 1, 2, and 3, respectively; and (4) the cumulative percentage of articles in each category in the last 3, 5, and 10 years. 

**Figure 4:**
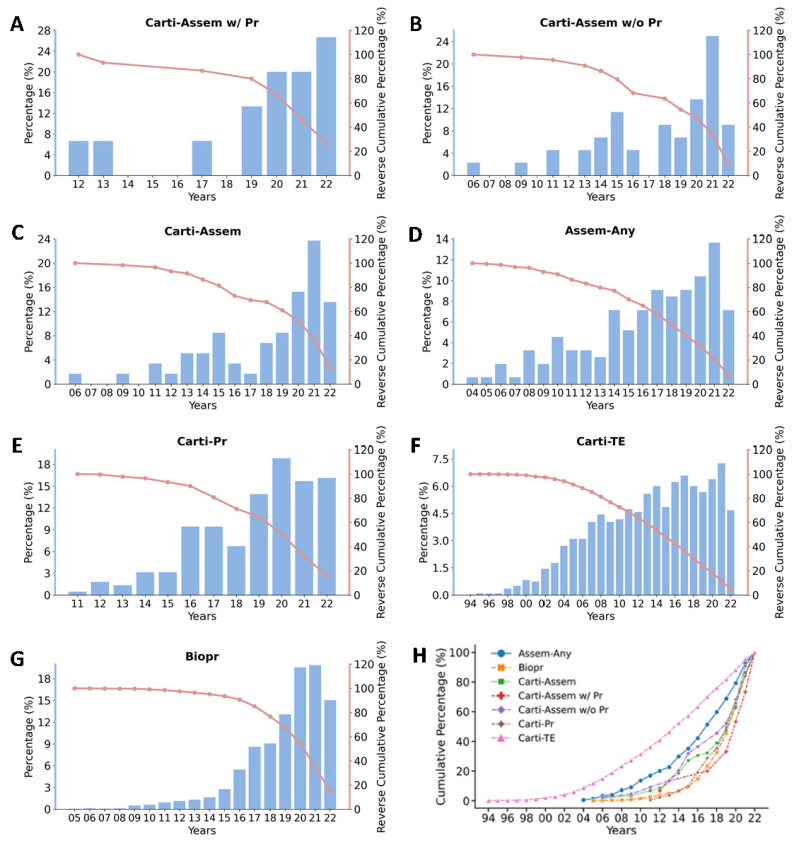
Analysis of the year trends of publication of seven categories in cartilage bioassembly, bioprinting, and tissue engineering. (**A**–**G**) Bar plots demonstrate the percentage of articles published each year. Line plots demonstrate the reverse cumulative percentage of articles published in each category. (**H**) Line plots demonstrate the cumulative percentage of articles in each category published in the last 28 years. Abbreviations: “Carti-Assem w/Pr”: micro-cartilage bioassembly with bioprinting; “Carti-Assem w/o Pr”: micro-cartilage bioassembly without bioprinting; “Carti-Assem”: Micro-cartilage bioassembly using any technologies; “Assem-Any”: bioassembly of any tissues; “Carti-Pr”: cartilage bioprinting; “Carti-TE”: cartilage tissue engineering; “Biopr”: bioprinting.

As shown in Table 3 and Figure 4, micro-cartilage bioassembly with bioprinting is the youngest and fastest-growing technology. It passed its first quartile in seven years, and 80% of its articles were published in the last three years. The first report of cartilage bioassembly without bioprinting (in 2006 [68]) is only two years behind the first bioassembly report (in 2004 [96]) using non-chondrogenic cells. The first report of cartilage bioassembly with bioprinting (in 2012 [86]) was eight years after the first bioassembly report and only one year after the first cartilage bioprinting (in 2011). The cumulative percentage plots (Figure 4H) demonstrate similar trends—that the cartilage bioassembly with bioprinting category has the steepest slope while the cartilage tissue engineering category has the flattest slope.

**Figure 5:**
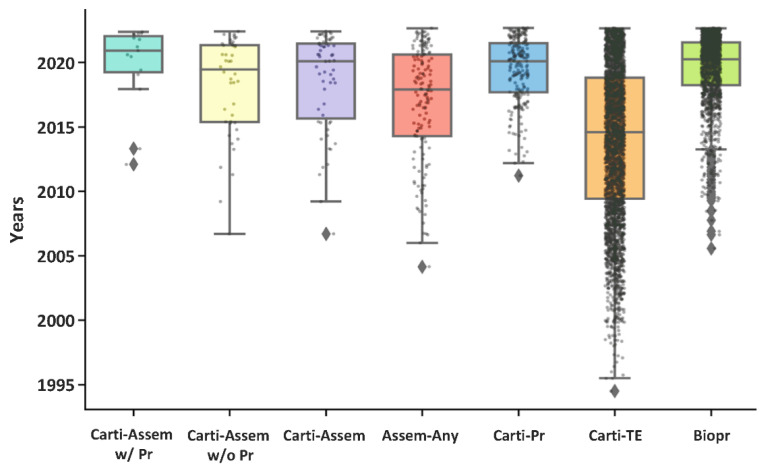
Box and strip plots of articles in each category. In each box, the top and bottom represent the first (Q1) and third (Q3) quartiles. The horizontal line inside the box is the median. Between Q1 and Q2 is the interquartile range (IQR), representing 50% of the articles. The whiskers extend 1.5 times the IQR beyond the top and bottom of the box, and outliers (diamonds) are beyond the whiskers. Abbreviations: “Carti-Assem w/Pr”: micro-cartilage bioassembly with bioprinting; “Carti-Assem w/o Pr”: micro-cartilage bioassembly without bioprinting; “Carti-Assem”: Micro-cartilage bioassembly using any technologies; “Assem-Any”: bioassembly of any tissues; “Carti-Pr”: cartilage bioprinting; “Carti-TE”: cartilage tissue engineering; “Biopr”: bioprinting.

Cartilage was one of the earliest research fields in tissue engineering, and the first report in the Pubmed database was in 1994 [97]. However, the first application of bioprinting in cartilage (reported in 2011 [98]) was six years behind the first application of bioprinting of any tissues. With 154 articles found in bioassembly application in any tissues, 59 articles were found in cartilage bioassembly, which is over 38% of the total bioassembly applications. The quartiles and the distributions of articles over the years in each category can be visualized in Figure 5, demonstrating that research in all the seven categories has been fast-developing in recent years. The micro-cartilage bioassembly and its correlated bioprinting technology are especially in a robust growing stage.

Cartilage tissue is composed of only chondrocytes, and this simple cell type distribution makes cartilage more suitable for bioassembly application, which may account for the fact that a large proportion of research on bioassembly has been targeting cartilage. However, with decades of research on cartilage regeneration, it has been found that the simplicity of cartilage makes it even harder to recreate [99]. We will discuss the existing problems and future directions in the following section.

## 7. Existing Problems

Even though research has made encouraging progress and one product using autologous chondrocyte spheroids has been licensed to repair articular cartilage defects [100], two critical issues still need to be solved. One issue is that chondrocytes tend to lose their native phenotype, changing to fibroblast-like cells. For decades, the challenge of preserving chondrocyte native phenotype in cartilage tissue engineering has remained. This issue also remains in the existing cartilage microtissue models, which negatively affects both individual microtissues and the assembled macro-tissue.

The native morphology of typical articular cartilage can hardly form in vitro in any type of building blocks, even though some positive biochemical or immunochemical staining can be seen. For scaffold-free cellular spheroids and cell-laden hydrogel microspheres, chondrocytes tend to lose their phenotypes, changing to fibroblastic type when chondrocytes are packed together. In cell-laden polymer microspheres, because the degradation of polymers occurs in vivo, cartilage-like tissue can only be seen in vivo in some areas of the assembled macro-tissue after the polymers degrade and neo tissues are remodeled. Native-like morphology cannot form in the individual microspheres either. With this limitation, improvement is needed for the existing cartilage microtissues before they can be reliably used as in vitro cartilage models.

Because of the above drawbacks in individual building blocks, the second issue is that the complete integration of the micro-cartilage building blocks into host cartilage is difficult to achieve. In previous reports, cellular spheroids were only able to fuse into a small and thin (<1.5 mm in the long axis, <0.75 mm in height) cartilage-like tissue [15]. When assembling a larger macro-cartilage tissue (>5 mm in diameter or equivalent circular diameter), boundaries of individual modules remain, necrotic cores form, and the mechanical properties of fused tissues are poor.

## 8. Future Directions and Conclusions

Regardless of the methods utilized to generate the building blocks in the previous reports, the cells within building blocks are usually homogenously packed in cellular spheroids or nearly evenly distributed in polymer or hydrogel microspheres. In contrast, cells usually distribute in certain patterns in their native tissues. Chondrocytes are organized in isogenous groups in native articular cartilage. These patterns occupy the initiation of articular cartilage and exist during the lifetime of healthy cartilage. Studies have demonstrated that patterns can serve as biophysical signals to control cell phenotype and functions [101,102]. Future studies may be inspired by the successful cellular patterning in other studies to pattern chondrocytes in a certain format inside building blocks. This may help individual building blocks grow into micro-cartilage with more native characteristics and help the integration among building blocks.

In conclusion, regenerating articular cartilage through bioassembly of tissue-engineered micro-cartilage building blocks is a promising technology. It has been rapidly developing in recent years, and we anticipate seeing more new technologies developed in this field and more pre-clinical and clinical applications reported.

## Figures and Tables

**Table 1:** Summary of the non-bioprinting bioassembly methods.

Bioassembly Method	Building Block Type	Building Block Generation Method	Cell Source	In Vivo
simple fusion [12,18,29,44,45,46,47,48,49,50,51,52,53,54,55,56,57,58,59]	aggregates [12,18,29,44,45,46,47,48,49,50,51,52,53,54,55,56]; cell-laden polymer microspheres [57,58]; cell-laden hydrogel microspheres [59]	multiple well plate [18,44,45,46,47,56]; micro-mold [48,50]; microwell [29,49,51,52,53,54,55]; spinner flask [12]; hanging drop [56]; phase separation [57]; water-in-oil-in-water (w/o/w) double emulsion method [58]; water-in-oil emulsion method [59]	chondrocytes [12,18,44,45,46,47,48,49,50,51,52,53,56,57,58]; ASCs [54,55]; MSCs [29,59]	mice [56,57]
mold [24,35,60,61,62,63,64,65,66,67,68]	aggregates [24,60,61,62,63,67,68]; cell-laden polymer microsphere [35,64,65,66]	96 well plate [24,60,63,67]; aggregate formation within hydrogels [61]; hanging drops [68]; microwell [62,64]; polymer self-assembly [35]; phase separation [65]; microfluidic process [66]	MSCs [24,60,61,63,64,66]; PDCs [62]; chondrocytes [35,65,68]; ASCs [67]	mice [35,62]; rabbit [35,63,64]; porcine [67]
multiple layers fusion [13,31]	aggregates [13,31]	microwell [13,31]	MSCs [13]; PDCs; iPSCs; chondrocytes [31]	mice [31]
graft [24,32,62,63,69,70]	aggregates [24,62,63,70]; cell-laden polymer microsphere [69]; cell-laden hydrogel microspheres [32]	multiple well plate [24,63]; microwell [62]; water in oil emulsion method [69]; conical tube [32]; hanging drops [70]	MSCs [24,63]; PDCs [62]; ASCs [69]; chondrocytes [32]; CSPCs [70]	mice [62,69]; rabbit [63,69]; canine [32]; rat [70]
defect filling [13,16,24,32,33,35,63,64,69,70,71,72,73,74,75]	aggregates [13,16,24,33,63,70,71,72,73]; cell-laden polymer microspheres [35,64,69,74,75]; cell-laden hydrogel microspheres [32]	multiple well plate [24,63,71]; hanging drops [70]; microwell [13,64]; microwell-mesh [16]; rotary shaker [33]; spinner flask [72]; suspension culture [73]; water in oil emulsion method [69,74]; polymer self-assembly [35]; conical tube [32]; water-in-oil-in-water (w/o/w) double emulsion method [75]	MSCs [16,24,63,64,74,75]; chondrocytes [13,32,33,35,71,72]; CSPCs [70]; ASCs [69]; PDC, iPSCs [13]; PSCs [73]	rabbit [33,35,63,64,69,74]; mice [13,16,35,69,71,73]; rat [70,75]; canine [33]

induced pluripotent stem cells (iPSCs); pluripotent stem cells (PSCs); cartilage stem/progenitor cells (CSPCs); periosteum derived cells (PDCs); mesenchymal stem/stromal cells (MSCs); adipose tissue derived stem cells (ASCs).

**Table 2:** Summary of the bioprinting bioassembly methods.

Bioassembly Method	Building Block Type	Building Block Generation Method	Cell Source	In Vivo
extrusion bioprinting [76,77,78]	aggregates [76,77,78]	microwell [76,77]; porous hydrogel [78]	MSCs [76]; chondrocytes [77]; ASCs [78]	-
aspiration-assisted bioprinting [79,80]	aggregates [79,80]	96 well plate [79,80]	MSCs [79]; ASCs [80]	-
spherical building blocks and polymer hybrid bioprinting [17,42,81,82,83,84,85,86,87]	aggregates [17,42,81,82,84,86,87]; cell-laden hydrogel microspheres [83,85]	96 well plate [17,81,84,86,87]; inkjet bioprinting in microchamber [42,82]; centrifuge tube [83,86]; microfluidic device [85]	ASCs [81,83]; MSCs [42,82,84,85]; chondrocytes [17,83,84,86,87]	rabbit [81]; mice [83]
needle-array-based spheroid bioassembly bioprinting [88]	aggregates [88]	96 well plate [88]	IPFP cells; MSCs [88]	rabbit [88]

infrapatellar fat pad (IPFP) cells; mesenchymal stem/stromal cells (MSCs); adipose tissue derived stem cells (ASCs).

**Table 3:** Summary of Milestones in the Literature Analysis.

	Carti-Assem w/Pr	Carti-Assem w/o Pr	Carti-Assem	Assem-Any	Carti-Pr	Carti-TE	Biopr
Number of Articles	15	44	59	154	223	3402	1590
1st-Pub. yr.	2012	2006	2006	2004	2011	1994	2005
Yrs. ≥ Q1 (Per., Date)	7 yrs. (33.3%, 2019)	9 yrs. (31.8%, 2015)	9 yrs. (27.1%, 2015)	11 yrs. (29.9%, 2014)	6 yrs. (28.7%, 2017)	15 yrs. (17.3%, 2009)	13 yrs. (32.5%, 2018)
Yrs. ≥ Q2 (Per., Date)	8 yrs. (53.3%, 2020)	13 yrs. (52.3%, 2019)	14 yrs. (62.7%, 2020)	14 yrs. (51.3%, 2017)	9 yrs. (68.2%, 2020)	20 yrs. (52.4%, 2014)	15 yrs. (65.1%, 2020)
Yrs. ≥Q3 (Per., Date)	10 yr. (100%, 2022)	15 yr. (90.9%, 2021)	14 yr. (86.4%, 2020)	17 yrs. (79.2%, 2020)	10 yrs. (83.9%, 2021)	24 yrs. (76%, 2018)	16 yrs. (85%, 2021)
Last 3 yrs. Cum. Per.	80%	54.3%	61%	40.3%	64.6%	24%	67.5%
Last 5 yrs. Cum. Per.	86.7%	63.6%	69.5%	57.8%	80.7%	36.6%	85.2%
Last 10 yrs. Cum. Per	100%	90.9%	93.2%	83.1%	99.6%	63.8%	97.5%

Abbreviations: “Carti-Assem w/Pr”: micro-cartilage bioassembly with bioprinting; “Carti-Assem w/o Pr”: micro-cartilage bioassembly without bioprinting; “Carti-Assem”: Micro-cartilage bioassembly using any technologies; “Assem-Any”: bioassembly of any tissues; “Carti-Pr”: cartilage bioprinting; “Carti-TE”: cartilage tissue engineering; “Biopr”: bioprinting; “Q1, Q2, Q3”: first, second, third quartile; “Pub.”: Publication; “Cum. Per.”: Cumulative Percentage.

## Data Availability

Not applicable.
